# Supplementary material for: Contact Patterns among High School Students
Source: PLoS One. 2014 Sep 16;9(9):e107878. doi: 10.1371/journal.pone.0107878 (PMC4167238; doi:10.1371/journal.pone.0107878)
Supplement: File S1 — File containing all supplementary figures and tables. (PDF) [file pone.0107878.s001.pdf]

Contact patterns among high-school students: Supplementary Text

J. Fournet[1] and A. Barrat[1,2]

[1] *Aix-Marseille Université, Université de Toulon, CNRS, CPT UMR 7332,13288 Marseille, France*

[2] *Data Science Laboratory, Institute for Scientific Interchange (ISI) Foundation, Torino, Italy*

DISTRIBUTION OF EDGE WEIGHTS INSIDE CLASSES AND BETWEEN CLASSES

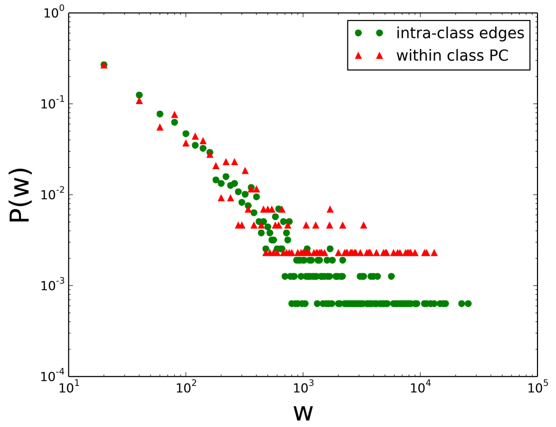

(a)

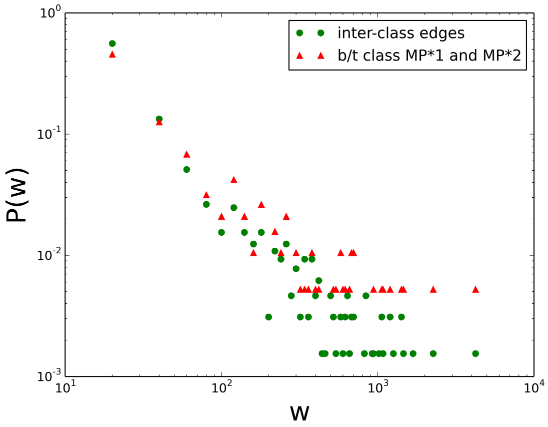

(b)

FIG. S1. Distribution of edge weights in the contact network aggregated over the whole study duration: (a) for all intra-class edges and for the edges between students of one specific class, (b) for all inter-class edges and for the edges between students of two specific classes.

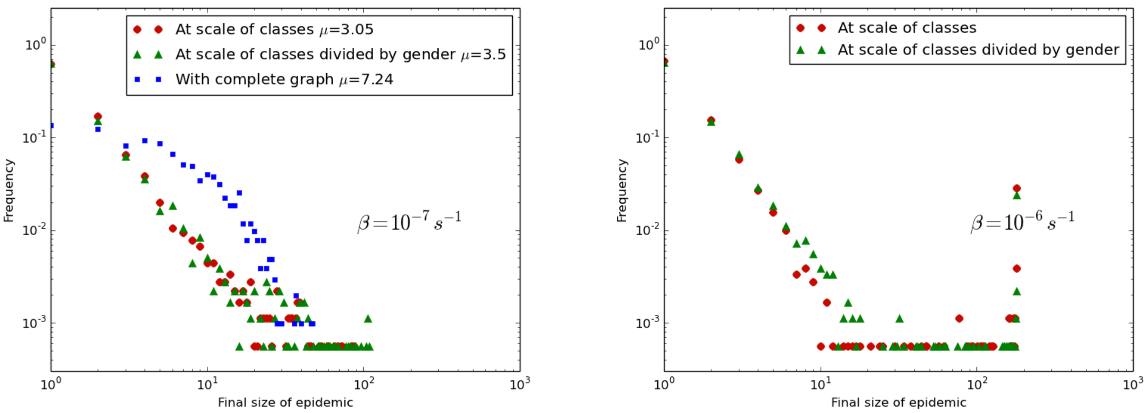

FIG. S2. Resuls of the simulation of a stochastic SI process with two different values for β using different representations of the contact patterns between students. Each plot shows the distribution of the number of cases after 10 hours (the duration of one day at school). Red symbols correspond to simulations using the contact matrix in which students are divided by classes, while the green symbols correspond to the result of simulations in which students are additionally divided according to gender. The blue symbols correspond to the results obtained under a homogeneous mixing assumption (not shown for the right plot as, for this value of β, all simulations lead then to having all individuals infected at the considered time.)

NUMERICAL SIMULATIONS OF A SIMPLE SPREADING PROCESS

Here we compare the spreading patterns of a simple stochastic Susceptible-Infected model among the students using two different representations of their contact patterns: (i) the first representation is given by the contact matrix of contact durations at the level of classes; (ii) the second representation uses a finer substructure in which each class is divided into two groups according to the students' gender, and is therefore given by the contact matrix of contact durations at the level of gender groups inside each class (see main text). In each case, each student of a group X is connected to all the students of another group Y with edges whose weight is given by $w_{XY}/(n_X n_Y)$. For reference, we also consider a homogeneous mixing representation in which each student is connected with all other students with a uniform weight equal $w = \sum_{ij} w_{ij}/(N(N-1))$ (i.e., we assume only the knowledge of the total aggregated time of all contacts between students, and assume that all pairs of students have spent the same time in contact). In each case, each Susceptible individual connected to an Infected individual by a link of weight w can become infected with rate βw (probability per unit time), where β is the spreading parameter of the model.

In order to compare the outcomes of the spreading processes, we show in Figure S2 the distribution of the number of infected individuals after a time of 10 hours, for two different values of β. The results are superimposed for the two contact matrix representations, while the homogeneous mixing representation of the data leads to a very different distribution, with a much larger average number of cases. This indicates that, at least for these parameters, a description of the contact patterns at the level of classes correspond to a sufficient resolution, and that the potential gender homophily does not make it necessary to represent the students population at the finer level of a division into gender groups in each class.

LONGITUDINAL STUDY

(a)

Cosine similarity b/t contacts matrices	1st Mon	1st Tues	Wed	Thurs	Fri	2nd Mon	2nd Tues
1st Mon	1	0.93	0.82	0.91	0.94	0.94	0.91
1st Tues	0.93	1	0.78	0.97	**0.99**	0.96	0.97
Wed	0.82	0.78	1	**0.65**	0.71	0.7	0.7
Thurs	0.91	0.97	**0.65**	1	**0.99**	0.96	0.96
Fri	0.94	**0.99**	0.71	**0.99**	1	0.97	0.97
2nd Mon	0.94	0.96	0.7	0.96	0.97	1	**0.99**
2nd Tues	0.91	0.97	0.7	0.96	0.97	**0.99**	1

(b)

Cosine similarity b/t contacts matrices	1st Mon	1st Tues	Wed	Thurs	Fri	2nd Mon	2nd Tues
1st Mon	1	0.78	0.77	0.79	0.86	0.84	0.88
1st Tues	0.78	1	**0.97**	0.66	0.96	0.90	0.65
Wed	0.77	**0.97**	1	0.68	0.96	0.92	**0.56**
Thurs	0.79	0.66	0.68	1	0.83	0.84	0.71
Fri	0.86	0.96	0.96	0.83	1	0.96	0.70
2nd Mon	0.84	0.90	0.92	0.84	0.96	1	0.66
2nd Tues	0.88	0.65	**0.56**	0.71	0.70	0.66	1

TABLE S1. (a) Cosine similarities between contacts matrices of the first column of Figures S3 and S4 (1st column). (b) Cosine similarities between contacts matrices in which diagonal elements are ignored. The numbers in blue (resp. red) are the maxima (resp. minima) of the table. The cosine similarity between 2 matrices M and N is defined as $\sigma(M,N) = \frac{\Sigma_{i,j} m_{ij} n_{ij}}{\sqrt{\Sigma_{i,j} m_{ij}^2}\sqrt{\Sigma_{i,j} n_{ij}^2}}$

Cosine similarity b/t morning and afternoon	
1st Monday	0.90
1st Tuesday	0.94
Wednesday	0.83
Thursday	0.53
Friday	0.73
2nd Monday	0.93
2nd Tuesday	0.94

TABLE S2. Cosine similarities between each pair of morning-afternoon contacts matrices of Figures S3 and S4 (2nd and 3rd columns).

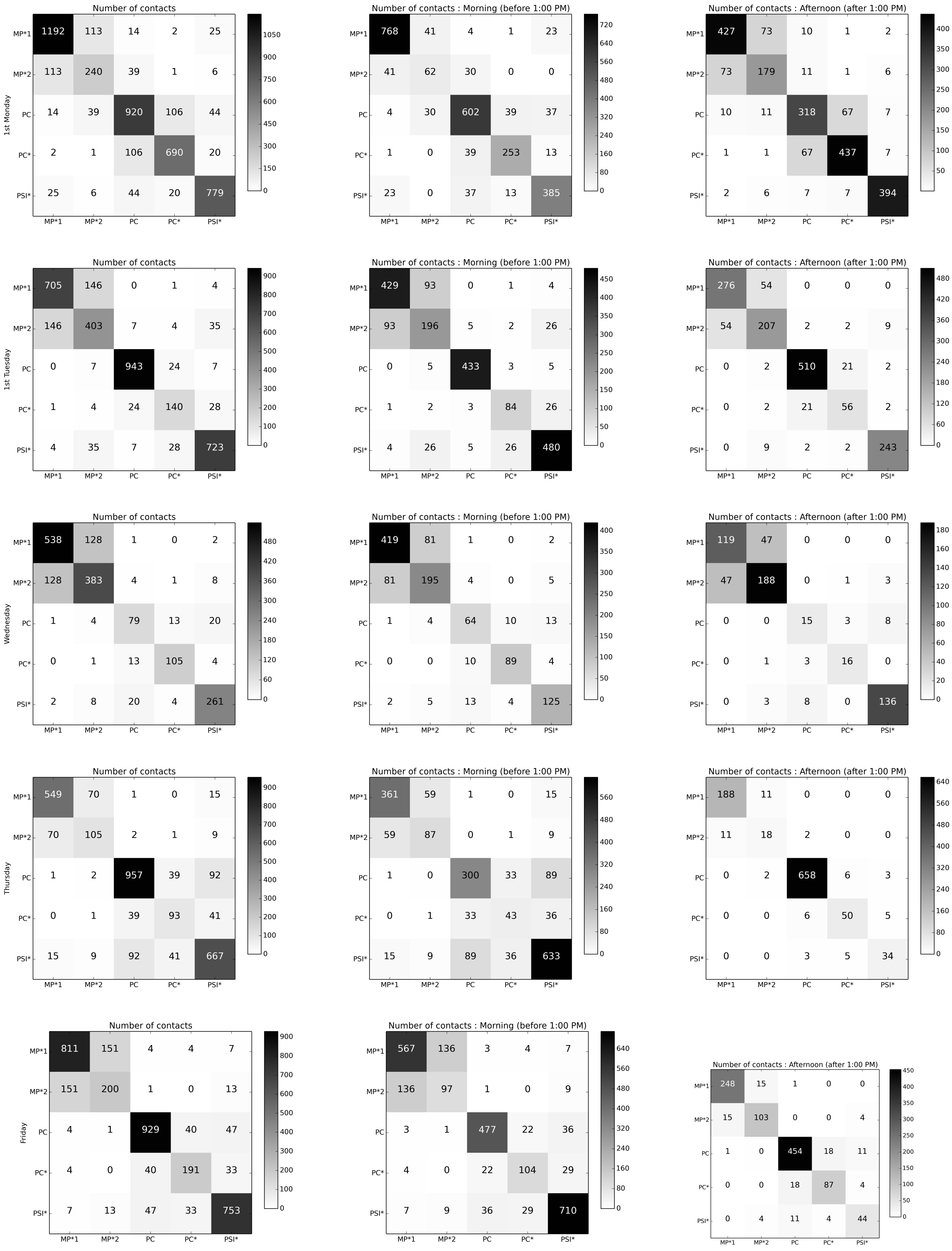

FIG. S3. First column: Contacts matrices giving the numbers of contacts between classes for each day of the first week. Second column: contact matrices for each morning (before 1:00 PM). Third column: same for each afternoon (after 1:00 PM). The matrix entry at row X and column Y gives the total number of contacts between all individuals of class X with all individuals of class Y during the aggregation interval (one day, one morning or one afternoon).

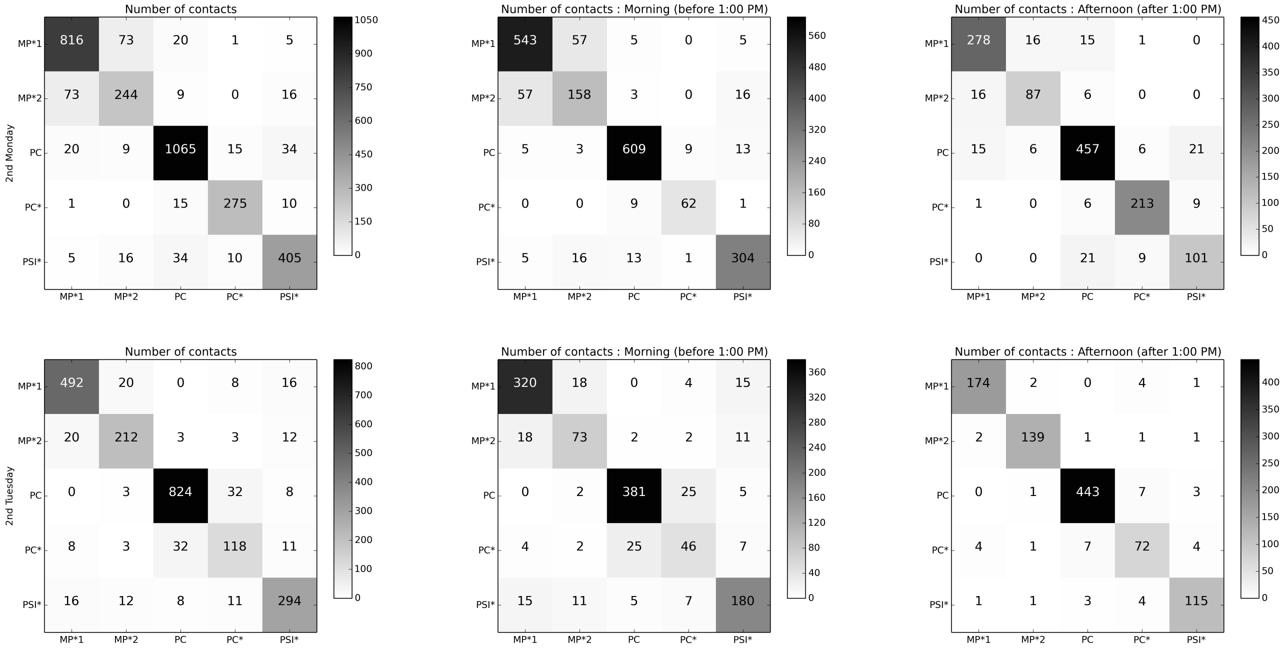

FIG. S4. Same as Figure S3 for the last two days.

Average cosine similarity b/t days of the study	1st Mon	1st Tues	Wed	Thurs	Fri	2nd Mon	2nd Tues
1st Mon	1	0.349	0.312	0.366	0.365	0.356	0.313
1st Tues	0.349	1	0.31	0.364	0.37	0.364	0.329
Wed	0.312	0.31	1	0.322	0.324	**0.292**	0.3
Thurs	0.366	0.364	0.322	1	**0.441**	0.356	0.302
Fri	0.365	0.37	0.324	**0.441**	1	0.349	0.347
2nd Mon	0.356	0.364	**0.292**	0.356	0.349	1	0.373
2nd Tues	0.313	0.329	0.3	0.302	0.347	0.373	1

TABLE S3. Average cosine similarities of the neighborhoods of nodes in the contact networks of different days. For the table entry at row X and column Y, we calculate the cosine similarity of the neighborhoods of each node in the contact networks aggregated over day X and day Y and take the average on all these similarities. The numbers in boldface are the maximum and minimum. These numbers need to be compared to the ones obtained with null models, given in Tables V, VI and VII.

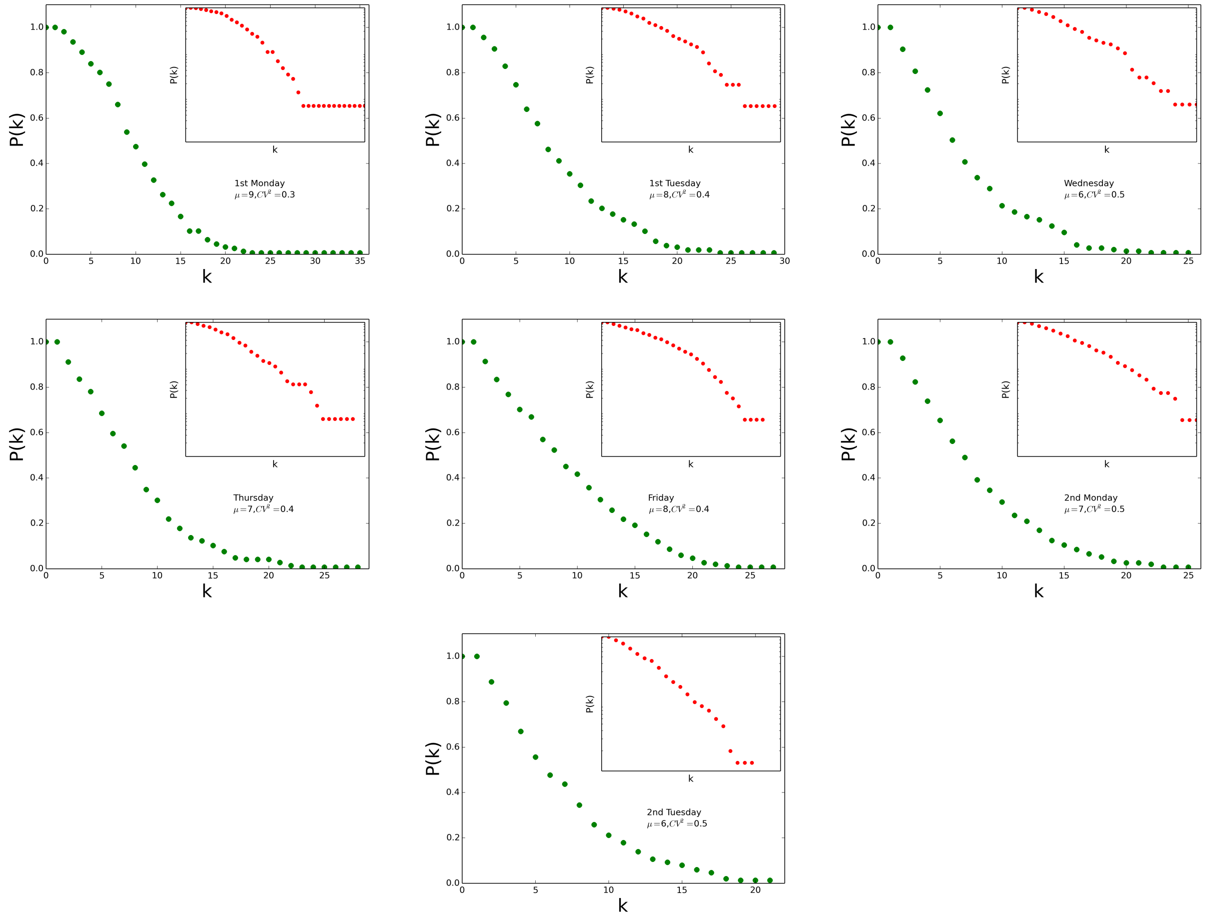

FIG. S5. Cumulative degree distribution $P(k)$ for the daily aggregated contact networks. The insets show the same distributions in lin-log scale.

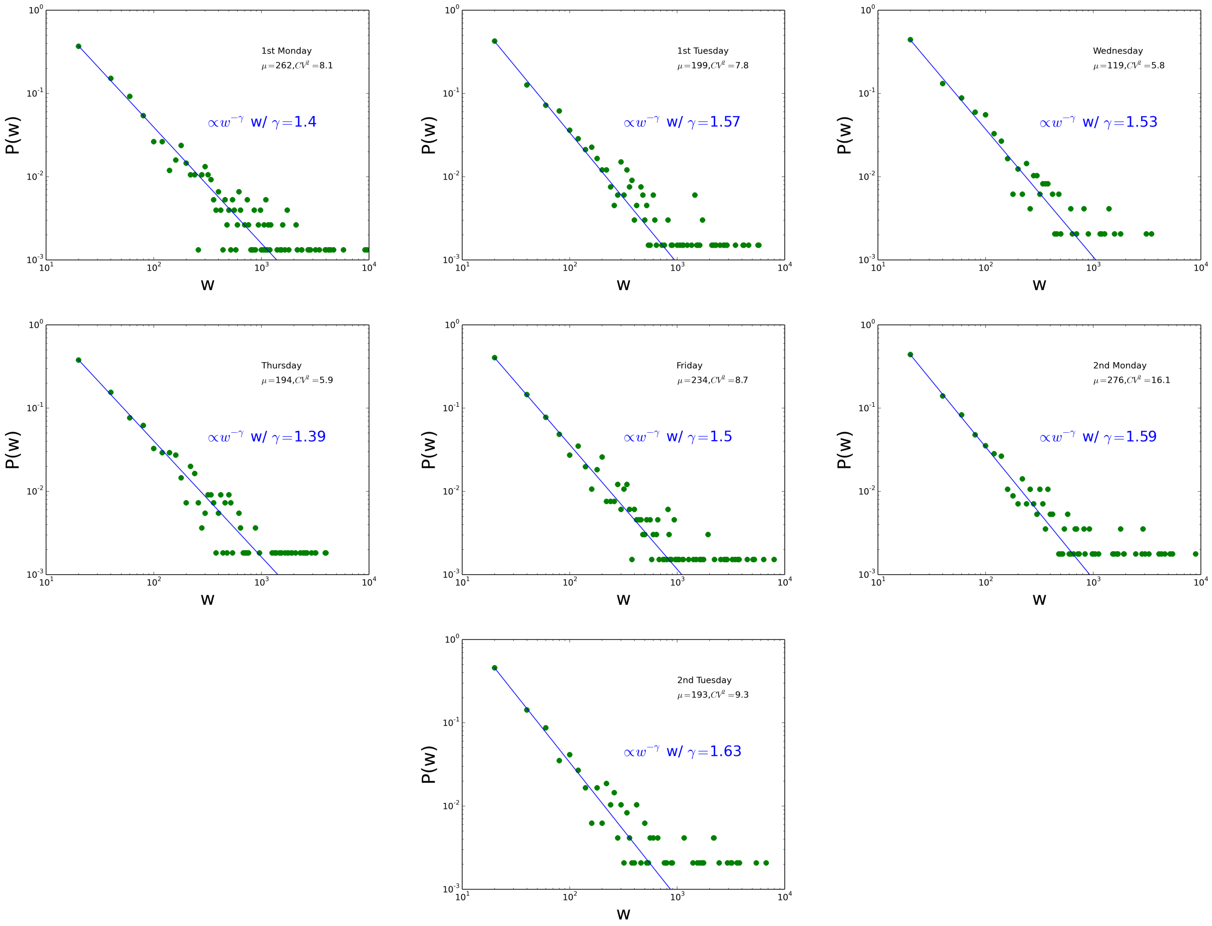

FIG. S6. Distribution of the edge weights for each daily aggregated contact networks.

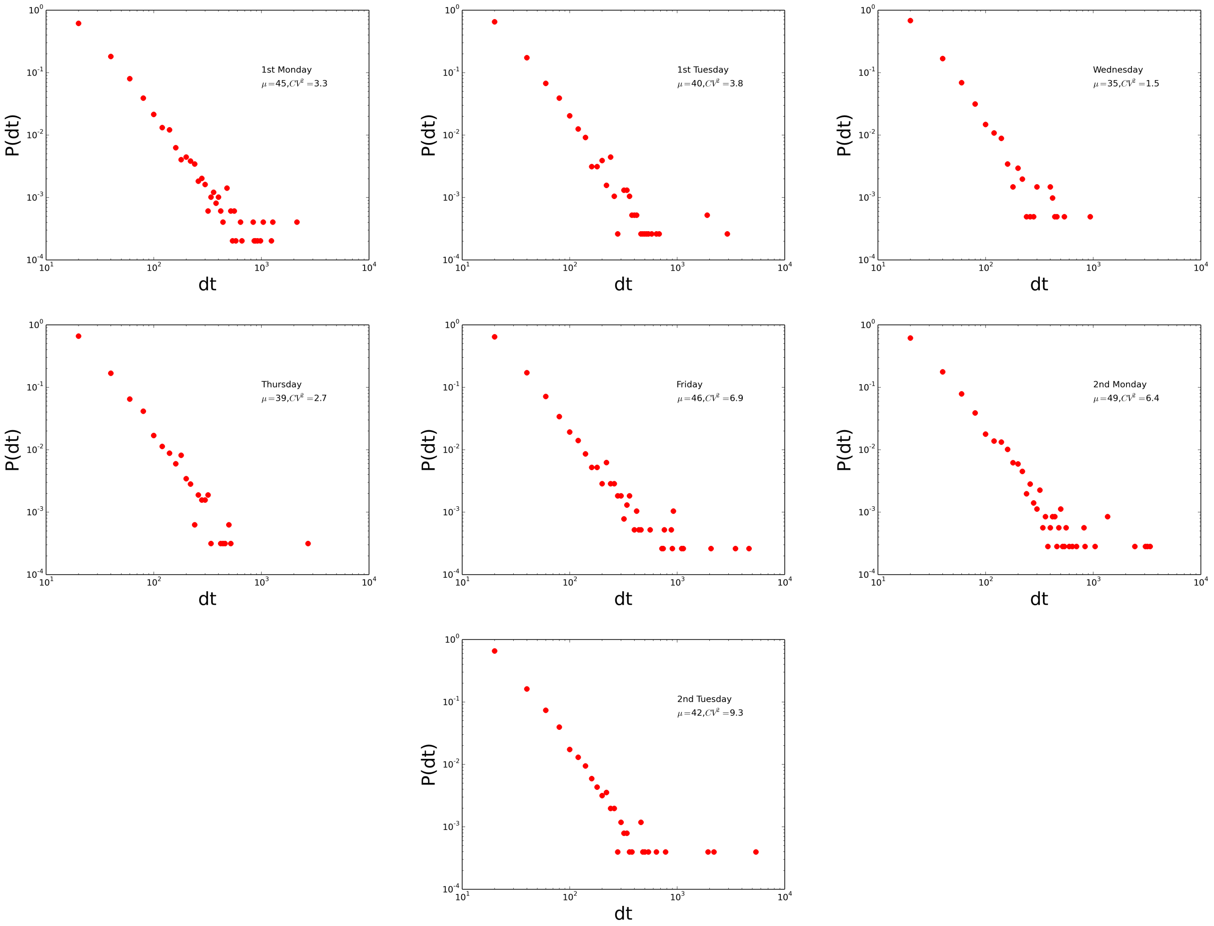

FIG. S7. Distribution of contact durations for each day.

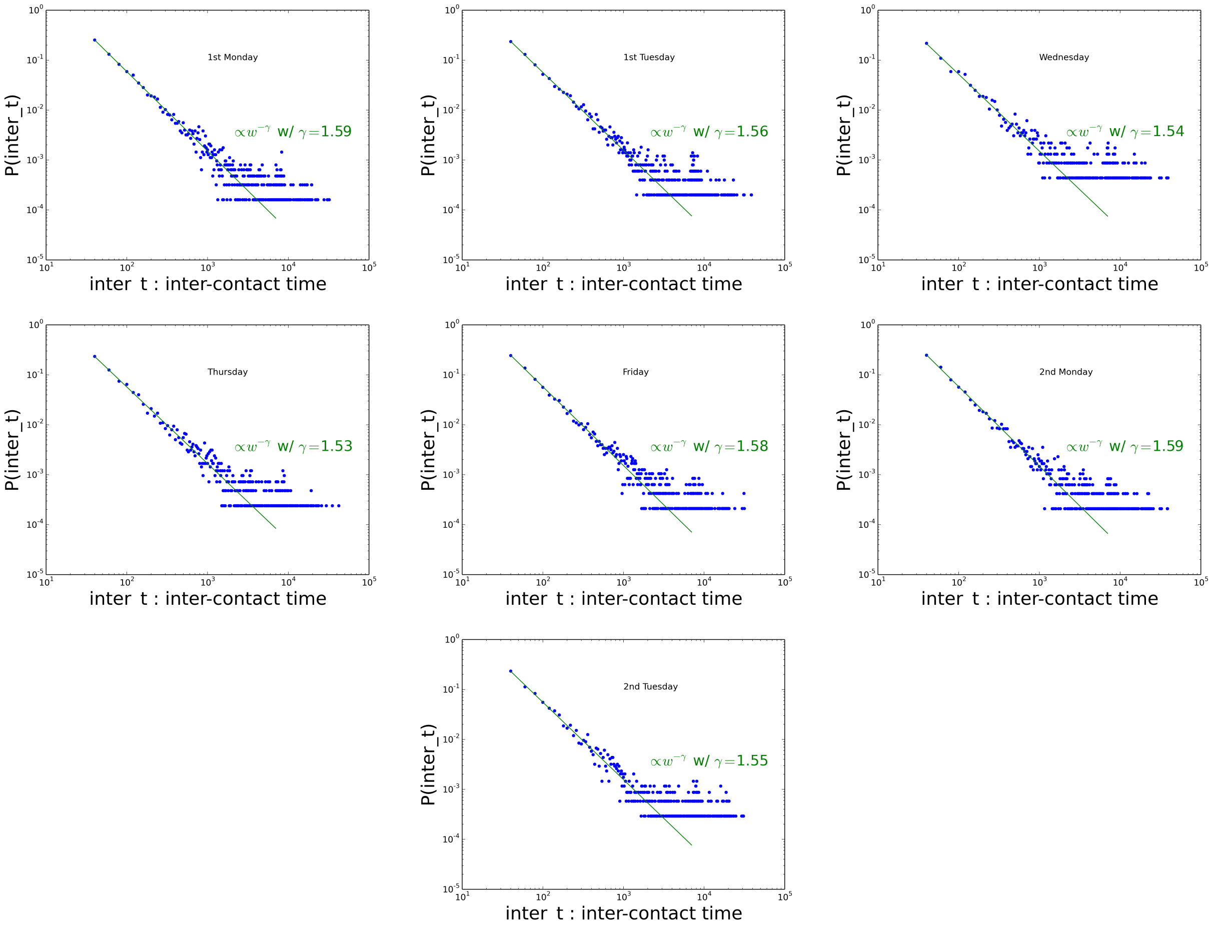

FIG. S8. Distribution of inter-contact durations for each day.

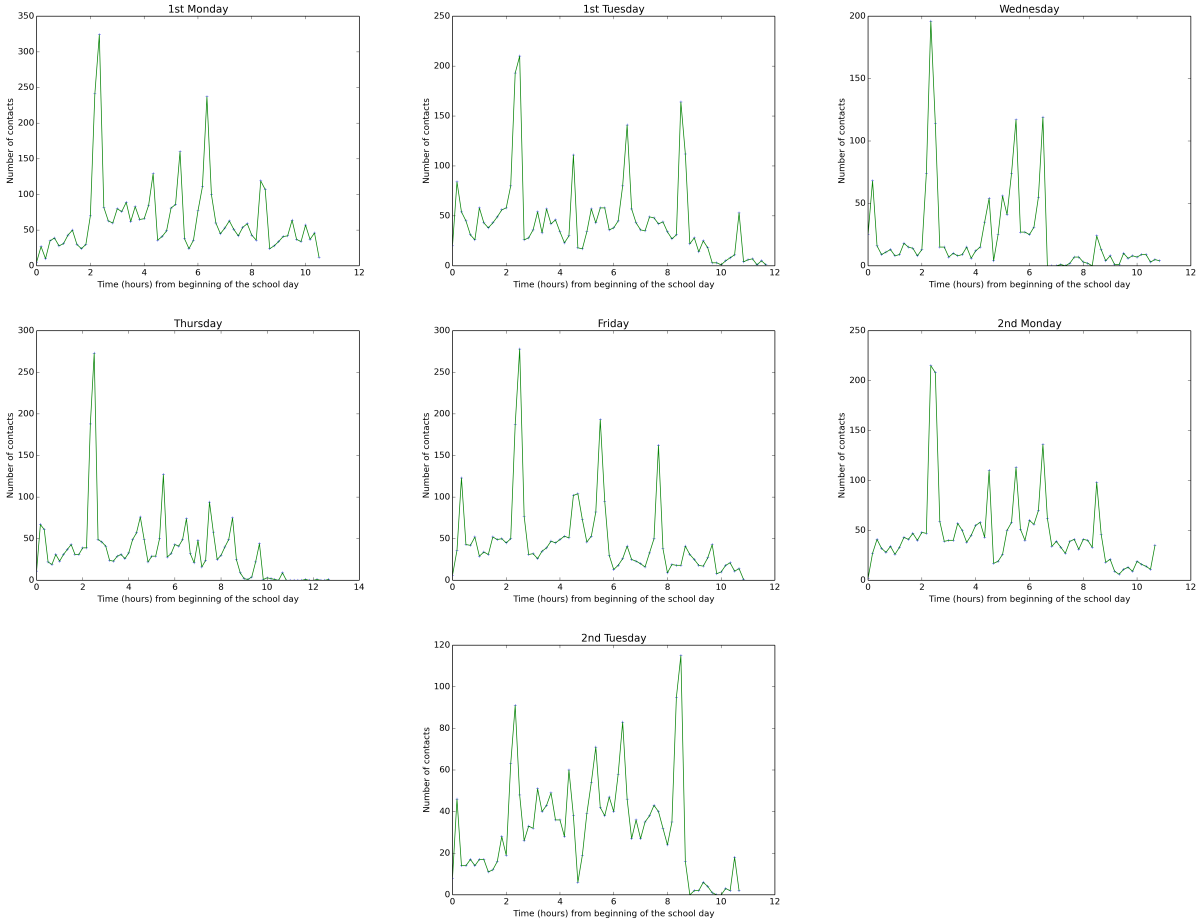

FIG. S9. Number of contacts per 10-minutes periods for each day.

Name of the null-model	Description
Rewiring (a)	All edges with their weight are replaced randomly in the graph.
Rewiring (b)	Same as above although each edge between class X and class Y is replaced randomly remaining between class X and class Y.
Rewiring Sneppen-Maslov (a)	Choose 2 edges A-B and C-D such that A is not linked to D and B is not linked to C; Remove these edges replacing them by edges A-D (with weight of edge A-B) and C-B (with weight of edge C-D). Repeat this procedure approximately $4 * E$ (E : number of edges) times. 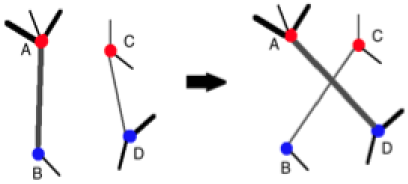
Rewiring Sneppen-Maslov (b)	Same as above although we do this separately for each pair of classes (and inside each class). Between two classes X and Y : A and C must be in class X and B and D must be in class Y. Inside a class X : A, B, C, D must be in class X. Repeat the procedure approximately $4 * E_{XY}$ (E_{XY}: number of edges between class X and class Y)/ $4 * E_{XX}$ (E_{XX}: number of edges inside class X) times.
Weight reshuffling (a)	The topology of the graph remains unchanged but the weights of the edges are reshuffled randomly.
Weight reshuffling (b)	Same as above although weight reshuffling is done for each pair of classes (or within each class) separately.

TABLE S4. Description of the null-models used.

(a)

Average cosine similarity b/t days of the study	1st Mon	1st Tues	Wed	Thurs	Fri	2nd Mon	2nd Tues
1st Mon	1.0	**0.016**	**0.016**	0.015	0.015	**0.014**	0.015
1st Tues	**0.016**	1.0	0.015	0.015	0.015	0.015	0.015
Wed	**0.016**	0.015	1.0	**0.014**	0.015	0.015	0.015
Thurs	0.015	0.015	**0.014**	1.0	0.015	0.015	0.015
Fri	0.015	0.015	0.015	0.015	1.0	0.015	0.015
2nd Mon	**0.014**	0.015	0.015	0.015	0.015	1.0	0.015
2nd Tues	0.015	0.015	0.015	0.015	0.015	0.015	1.0

(b)

Average cosine similarity b/t days of the study	1st Mon	1st Tues	Wed	Thurs	Fri	2nd Mon	2nd Tues
1st Mon	1.0	**0.017**	0.016	0.015	0.016	0.016	0.015
1st Tues	**0.017**	1.0	0.016	0.015	0.016	**0.017**	0.015
Wed	0.016	0.016	1.0	0.015	0.015	0.015	0.016
Thurs	0.015	0.015	0.015	1.0	0.015	0.015	0.014
Fri	0.016	0.016	0.015	0.015	1.0	0.015	0.016
2nd Mon	0.016	**0.017**	0.015	0.015	0.015	1.0	**0.013**
2nd Tues	0.015	0.015	0.016	0.014	0.016	**0.013**	1.0

TABLE S5. Average cosine similarities of nodes in pairs of synthetic daily contact networks obtained with the null-models ”Rewiring” (a) and (b) defined in Table IV. The numbers in boldface are the maximum and minimum.

(a)

Average cosine similarity b/t days of the study	1st Mon	1st Tues	Wed	Thurs	Fri	2nd Mon	2nd Tues
1st Mon	1.0	0.021	0.019	0.02	0.021	0.019	**0.017**
1st Tues	0.021	1.0	0.023	0.023	0.021	0.02	0.019
Wed	0.019	0.023	1.0	0.018	0.021	0.018	0.018
Thurs	0.02	0.023	0.018	1.0	0.023	**0.024**	0.021
Fri	0.021	0.021	0.021	0.023	1.0	0.02	0.023
2nd Mon	0.019	0.02	0.018	**0.024**	0.02	1.0	0.02
2nd Tues	**0.017**	0.019	0.018	0.021	0.023	0.02	1.0

(b)

Average cosine similarity b/t days of the study	1st Mon	1st Tues	Wed	Thurs	Fri	2nd Mon	2nd Tues
1st Mon	1.0	0.092	0.098	0.1	0.094	0.079	0.082
1st Tues	0.092	1.0	0.092	0.099	0.108	0.097	**0.078**
Wed	0.098	0.092	1.0	0.109	0.101	0.09	0.088
Thurs	0.1	0.099	0.109	1.0	**0.12**	0.095	0.086
Fri	0.094	0.108	0.101	**0.12**	1.0	0.096	0.081
2nd Mon	0.079	0.097	0.09	0.095	0.096	1.0	0.081
2nd Tues	0.082	**0.078**	0.088	0.086	0.081	0.081	1.0

TABLE S6. Average cosine similarities of nodes in pairs of synthetic daily contact networks obtained with the null-models ”Sneppen-Maslov rewiring” (a) and (b) defined in Table IV. The numbers in boldface are the maximum and minimum.

(a)

Average cosine similarity b/t days of the study	1st Mon	1st Tues	Wed	Thurs	Fri	2nd Mon	2nd Tues
1st Mon	1.0	0.142	0.14	0.125	**0.116**	0.117	0.122
1st Tues	0.142	1.0	0.151	0.153	0.129	0.132	0.125
Wed	0.14	0.151	1.0	0.149	0.145	0.129	**0.158**
Thurs	0.125	0.153	0.149	1.0	0.157	0.142	0.139
Fri	**0.116**	0.129	0.145	0.157	1.0	0.141	0.138
2nd Mon	0.117	0.132	0.129	0.142	0.141	1.0	0.164
2nd Tues	0.122	0.125	**0.158**	0.139	0.138	0.164	1.0

(b)

Average cosine similarity b/t days of the study	1st Mon	1st Tues	Wed	Thurs	Fri	2nd Mon	2nd Tues
1st Mon	1.0	0.153	0.154	0.135	**0.123**	0.13	0.134
1st Tues	0.153	1.0	0.16	0.167	0.145	0.144	0.137
Wed	0.154	0.16	1.0	0.165	0.16	0.144	0.175
Thurs	0.135	0.167	0.165	1.0	**0.179**	0.161	0.153
Fri	**0.123**	0.145	0.16	**0.179**	1.0	0.154	0.156
2nd Mon	0.13	0.144	0.144	0.161	0.154	1.0	0.182
2nd Tues	0.134	0.137	0.175	0.153	0.156	0.182	1.0

TABLE S7. Average cosine similarities of nodes in pairs of synthetic daily contact networks obtained with the null-models ”Weight reshuffling” (a) and (b) defined in Table IV. The numbers in boldface are the maximum and minimum.
